# Identification of Plakortide E from the Caribbean Sponge *Plakortis halichondroides* as a Trypanocidal Protease Inhibitor using Bioactivity-Guided Fractionation

**DOI:** 10.3390/md12052614

**Published:** 2014-05-02

**Authors:** Swarna Oli, Usama Ramadan Abdelmohsen, Ute Hentschel, Tanja Schirmeister

**Affiliations:** 1 Institute of Pharmacy and Biochemistry, Johannes Gutenberg-University of Mainz, Staudinger Weg 5, Mainz 55128, Germany; E-Mail: swarnoli@uni-mainz.de; 2 Department of Botany II, Julius-von-Sachs-Institute for Biological Sciences, University of Würzburg, Julius-von-Sachs Platz 3, Würzburg 97082, Germany; E-Mails: usama.ramadan@uni-wuerzburg.de (U.R.A.); ute.hentschel@uni-wuerzburg.de (U.H.); 3 Department of Pharmacognosy, Faculty of Pharmacy, Minia University, Minia 61519, Egypt

**Keywords:** *Plakortis halichondroides*, plakortide E, protease inhibitor, slowly-binding reversible inhibitor, cathepsin, rhodesain

## Abstract

In this paper, we report new protease inhibitory activity of plakortide E towards cathepsins and cathepsin-like parasitic proteases. We further report on its anti-parasitic activity against *Trypanosoma brucei* with an IC_50_ value of 5 μM and without cytotoxic effects against J774.1 macrophages at 100 μM concentration. Plakortide E was isolated from the sponge *Plakortis halichondroides* using enzyme assay-guided fractionation and identified by NMR spectroscopy and mass spectrometry. Furthermore, enzyme kinetic studies confirmed plakortide E as a non-competitive, slowly-binding, reversible inhibitor of rhodesain.

## 1. Introduction

Proteases enable breakdown of proteins via catalytic hydrolysis of peptide bonds [1]. Malfunction in the control of protease activity leads to undesired and unregulated proteolysis which causes many diseases. Therefore, inhibitors of proteases have the potential to provide successful therapeutics for a wide range of diseases [2,3].

**Figure 1:**
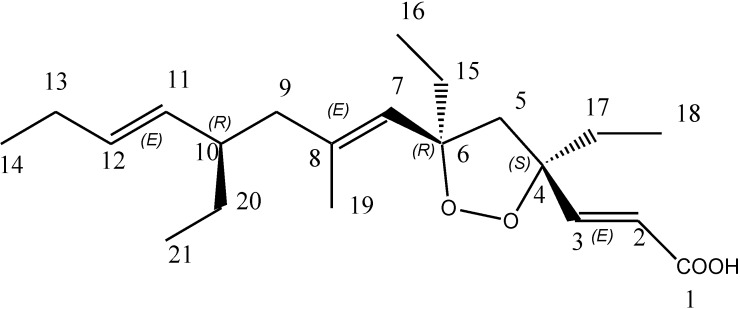
Structure of plakortide E.

Marine sponges of the family Plakinidae are known to be rich sources of structurally unique and biologically active metabolites [4]. Bioactivity-guided fractionation of the crude cyclohexane extract from the sponge *Plakortis halichondroides* yielded a pure endoperoxide metabolite, named plakortide E (Figure 1), which was previously isolated from the same sponge species [5,6]. Plakortide E was previously shown to stimulate sarcoplasmic reticulum (SR) Ca^2+^ ATPase activity [5]. Other endoperoxides from the plakortin family, e.g., six-membered plakortin [7], dihydroplakortin, 3-epiplakortin, plakortide Q [8] and plakortide M [9], are known to be active against Plasmodia, while the five-membered endoperoxide plakortide E was reported to be inactive [10]. In this work, we highlight its new anti-protease and anti-parasitic activities.

## 2. Results and Discussion

**Figure 2:**
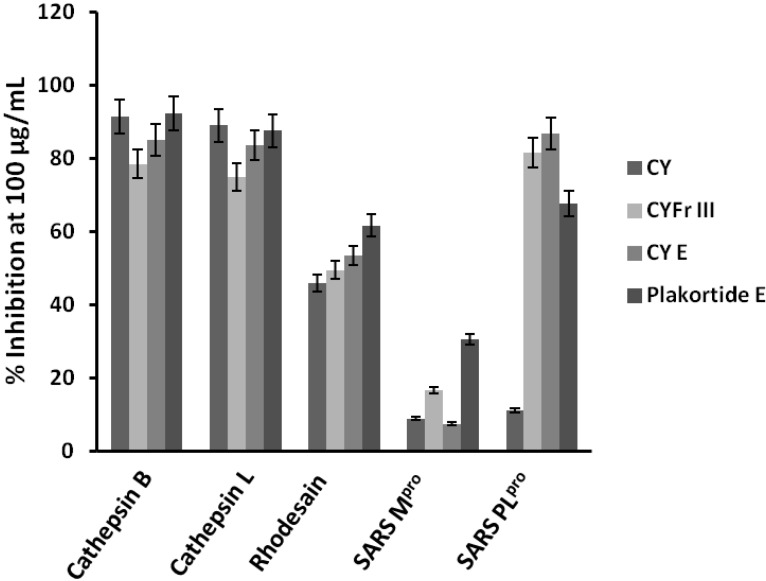
Protease inhibitory activity of the crude cyclohexane extract (CY), the subfractions CYFr III and CY E as well as plakortide E.

The lyophilized material of the sponge *Plakortis halichondroides* was sequentially extracted with three different solvents and the crude extracts were tested for protease inhibitory activity against the following proteases: Human cysteine proteases cathepsin B [11] and L [12], the related parasite enzyme rhodesain [13] from *Trypanosoma brucei rhodesiense*, and the two cysteine proteases expressed by the SARS coronavirus, namely SARS main protease [14] and SARS papain-like protease [15]. The active crude cyclohexane extract (CY) was further fractionated using column chromatography and finally purified with HPLC to yield the active pure metabolite plakortide E (Figure 1). The purification process was based upon the bioactivity results, *i.e.*, only fractions which showed activity against the enzymes were purified further. The activity of the fractions gradually enhanced with every step of purification process, with the exception of the first cyclohexane extract whichshowed very high inhibition of cathepsins B and L (Figure 2) probably being due to presence of other non polar active compounds.

**Figure 3:**
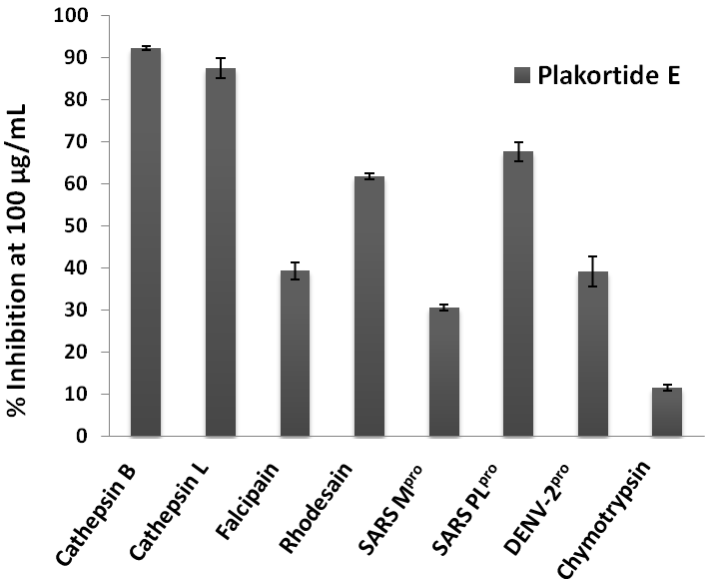
Percent inhibition of proteases by plakortide E at 100 μg/mL (285 μM), tests were performed in triplicates.

The pure metabolite plakortide E was further tested against the parasite cathepsin-L like protease falcipain-2 [16] from *Plasmodium falciparum*, as well as against the mammalian serine proteases chymotrypsin and the serine protease from Dengue virus (NS2B/NS3 protease) [17]. Inhibition at 100 μg/mL (285.71 μM) was only found with the cathepsins and the cathepsin-like proteases (Figure 3), especially with cathepsin B, L, rhodesain and SARS PL^pro^.

In order to study the inhibition mechanism of plakortide E, enzyme assays [18] with various inhibitor and substrate concentrations were performed with cathepsin B to determine whether the inhibition is competitive with respect to the substrate. The IC_50_ values for cathepsin B were determined at three different substrate concentrations (50, 100, 200 μM). The values (29, 26, 36 μg/mL, 82, 73, 103 μM) were almost similar indicating non-competitive inhibition. For cathepsin L, an IC_50_ of 37 μg/mL (105 μM) at a substrate concentration of [S] = 6.25 μM and for rhodesain an IC_50_ of 44 μg/mL (124 μM) at a substrate concentration of [S] = 30.0 μM was determined. With rhodesain, non-linear progress curves for the substrate hydrolysis were observed in the presence of inhibitor. Therefore, we determined the time-dependency of inhibition by measuring IC_50_ values in correlation to incubation time of enzyme with the inhibitor added, prior to substrate addition. The IC_50_ values decrease with longer incubation times (5 min → 90 μg/mL (257 μM); 30 min → 72 μg/mL (205 μM); 60 min → 27 μg/mL (77 μM)) indicating covalent inhibition or other mechanisms leading to slow binding. To address whether the inhibition is reversible or irreversible, further kinetic studies were performed as described by Copeland [19]. Dilution assays were performed and compared with K11777 [20], an irreversible vinylsulfone-based inhibitor of rhodesain. The enzyme rhodesain (100 fold concentration as used in the normal enzyme assays) was preincubated with the 10 fold IC_50_ concentration of the compound for one hour, which allows the formation of enzyme-inhibitor complexes and leads to total block of enzyme activity. The above complex was then diluted 100 fold by adding assay buffer and substrate. Thus, the enzyme concentration was reduced to the one used in the normal assays and the inhibitor concentration was reduced to 1/10 of the IC_50_. If reversible, the inhibitor will dissociate from the complex and enzyme activity is recovered. In case of irreversible inhibition, dissociation of the complex cannot occur and no enzyme activity will be detected. These assays showed reactivation of the enzyme in the case of plakortide E to about 50% activity, while in contrast no enzyme activity was detected with K11777. This indicates a reversible inhibition by plakortide E. In summary, plakortide E was determined to be a non-competitive, reversible, inhibitor of cysteine proteases, in case of rhodesain slow-binding was observed.

Furthermore, plakortide E was tested against the parasites *Leishmania major* promastigotes and the trypomastigote forms of *Trypanosoma brucei*, *Candida albicans*, and was also tested for its cytotoxicity against J774.1 macrophages. The compound exhibited trypanocidal activity with an IC_50_ value of 5 μM (after 48 h and also after 72 h). This may at least in parts be due to the protease inhibiting properties of plakortide E. It did not show activity against *Leishmania* which also express a variety of cathepsin-like proteases [21]. No activity against *Candida*, and no cytotoxic effects against macrophages at 100 μM were observed. Since *Leishmania* promastigote forms express less cysteine proteases than the amastigote forms, the cysteine-protease inhibiting properties of the compound may not be sufficient for detectable leishmanicidal activity.

## 3. Experimental Section

The sponge *Plakortis halichondroides* was collected by SCUBA diving at depths of 30 m in Bahamas in July 2008 (GPS: 26°27′3.25″N, 77°54′14.59″W). Sponge tissues were cut into small pieces and preserved at −80 °C until extraction. The frozen material was then dried by lyophilization. The lyophilized material (640 g) was subsequently macerated and sequentially extracted with cyclohexane (CY), methylene dichloride (DCM), and finally methanol (MeOH). After filtration, the crude extracts were concentrated under reduced pressure. The crude cyclohexane extract (15.27 g) was chromatographed on a silica gel (200 g) column and eluted with an isocratic solvent (cyclohexane/methylene dichloride/methanol/formic acid (2:1:1:0.05)). The eluted fractions were combined based upon TLC results to yield five fractions (CYFr I–V). Further fractionation of the fraction CYFr III by silica gel column chromatography using the solvent system (cyclohexane/methylene dichloride (90:10) with increasing polarity (chloroform/methanol (10:90)) afforded seven subfractions (CY A–G). The subfraction CY E was subjected to preparative HPLC using a RP 18 column (eluent methanol/water with 0.1% formic acid 70:30, flow 8 mL/min) affording 3 fractions (CY M, N and P). The fraction CY N was further purified using preparative HPLC using RP 18 column (methanol/water amended with 0.1% formic acid 70:30, flow 8 mL/min, and the retention time of the peak was observed at 40 min) to yield the pure bioactive compound **1**. The compound **1** was identified as plakortide E, by means of MS and NMR spectral data (Table 1) and comparison to previously published NMR data [5,6,22]. Enzyme assays [18,21,23,24,25] and parasite growth assays [21,23,24,25,26] were performed as described previously.

**Table 1:** NMR-spectroscopic data of plakortide E (**1**) in CDCl_3_ (^1^H: 400 MHz; ^13^C: 100 MHz, δ in ppm).

Position	δ_C_	Multiplicity	δ_H_	Multiplicity	*J* (Hz)
1	171.76	C			
2	119.75	CH	6.09	d	15.8
3	152.31	CH	6.96	d	15.8
4	87.31	C			
5	56.11	CH_2_	2.55	d	12.0
			2.45	d	12.0
6	89.39	C			
7	126.71	CH	5.12	s	
8	136.82	C			
9	46.67	CH_2_	2.00	m	
			1.85	m	
10	42.68	CH	1.99	m	
11	132.91	CH	5.05	ddt	15.2, 8.3
12	132.10	CH	5.34	dt	15.2, 6.3, 6.3
13	25.73	CH_2_	1.96	m	
14	14.19	CH_3_	0.94	t	7.4
15	32.36	CH_2_	1.88	m	
			1.64	m	
16	9.01	CH_3_	0.89	t	7.4
17	30.88	CH_2_	1.76	m	
18	8.95	CH_3_	0.92	t	7.5
19	17.92	CH_3_	1.62	d	1.2
20	27.79	CH_2_	1.34	m	
			1.10	m	
21	11.72	CH_3_	0.81	t	7.4

NMR spectra (Table 1) were obtained with a BRUKER (Bruker Biospin GmbH, Rheinstetten Germany), Typ Advance 400 spectrometer. Mass spectra were measured using a Bruker micrOTOF 88 mass spectrometer. Column chromatography was performed using silica gel (0.063–0.200 mm mesh, Merck, Darmstadt, Germany). TLC was conducted on pre-coated silica gel 60 F254 plates (0.20 mm, Merck, Darmstadt, Germany); spots were detected using vanillin spray reagent, UV 254 nm and iodinevapours. Reagents were purchased from Sigma-Aldrich (Munich, Germany) or Fluka (Munich, Germany). Solvents were purchased from Roth (Karlsruhe, Germany) or Merck (Darmstadt, Germany). High performance liquid chromatography was performed on a Varian ProStar (Rheinfelden, Schweiz), consisting of an analytical/preparative HPLC Upscale Linear system (0.05–50 mL/min at 275 bar pressure with scale-mast), a preparative autosampler and a 2-channel UV detector. The detection wavelengths were 254 nm and 230 nm.

Plakortide E: The product was obtained as colourless viscous oil (0.018 g). CDCl_3_ was used as solvent for measuring NMR spectra. ESI-MS found: 373.23404 [M + Na]^+^, calcd. for C_21_H_34_O_4_, 350.49. The specific rotation of plakortide E was [α]^d^_22_ = +60.7°, *c* = 0.00313 in CHCl_3_.

Enzyme assays and *in vitro* tests for antiparasitic activity were performed as published previously: for cathepsin-like cysteine proteases see [18,23,24,25,26], for SARS M^pro^ see [27], for SARS PL^pro^ see [15], for Dengue virus protease see [28], for assays against *T. brucei* see [24,29,30,31,32], for assays on macrophages see [33], for assays on *L. major* promastigotes see [21], for assays on *C. albicans* see [34,35].

## 4. Conclusions

Plakortide E, obtained from the marine sponge *Plakortis halichondroides*, was identified as a new protease inhibitor. Plakortide E showed selectivity towards the cathepsin-like cysteine proteases, with a non-competitive, reversible, and, in the case of rhodesain, a slow-binding inhibitory mode of action. The anti-protease activity of the compound may contribute to its anti-parasitic activity against *Trypanosoma brucei*, as rhodesain and also the cathepsin B like protease TbCatB [13] are known to be essential for the parasite’s growth and pathogenicity.
